# Supplementary material for: A polymer controlled nucleation route towards the generalized growth of organic-inorganic perovskite single crystals
Source: Nat Commun. 2021 Apr 1;12:2023. doi: 10.1038/s41467-021-22193-1 (PMC8016843; doi:10.1038/s41467-021-22193-1)
Supplement: Supplementary file 1 — Supplementary Information [file 41467_2021_22193_MOESM1_ESM.pdf]

A Polymer Controlled Nucleation Route towards the Generalized Growth of Organic-Inorganic Perovskite Single Crystals

Lin Ma[1,2], Zhengguang Yan[1,2]*, Xiaoyuan Zhou[3*], Yiqun Pi[1,2], Yiping Du[1,2], Jie Huang[1,2], Kaiwen Wang[1,2], Ke Wu[4], Chunqiang Zhuang[1,2], and Xiaodong Han[1,2]*

[1]Institute of Microstructure and Property of Advanced Materials, Faculty of Materials and Manufacturing, Beijing University of Technology, Beijing 100124, China.
[2]Beijing Key Laboratory of Microstructure and Properties of Solids, Beijing University of Technology, Beijing 100124, China.
[3]College of Physics and Institute of Advanced Interdisciplinary Studies, Chongqing University, Chongqing 401331, China.
[4]Beijing National Laboratory for Molecular Sciences, State Key Laboratory of Rare Earth Materials Chemistry and Applications, PKU-HKU Joint Laboratory in Rare Earth Materials and Bioinorganic Chemistry, College of Chemistry and Molecular Engineering, Peking University, Beijing 100871, China.
E-mail: yanzg@bjut.edu.cn; xiaoyuan2013@cqu.edu.cn; xdhan@bjut.edu.cn;

E-mail: yanzg@bjut.edu.cn; xiaoyuan2013@cqu.edu.cn; xdhan@bjut.edu.cn;

Supplementary Table 1. The sizes of the OIHPs single crystals in Figure 1, Main text

OIHPs single crystal	$FAPbI_3$	$FAPbBr_3$	$CsPbBr_3$
Size: L×W (mm×mm)	11×10	8×8	10×3
OIHPs single crystal	$MAPbBr_3$	$MAPbCl_3$	$MAPbI_3$
Size: L×W (mm×mm)	8×6	5×5	12×10
OIHPs single crystal	$MAPbI_{2.1}Br_{0.9}$	$MAPbI_{1.6}Br_{1.4}$	$MAPbI_{0.12}Br_{2.88}$
Size: L×W (mm×mm)	10×8	5×5	8×8
OIHPs single crystal	$MAPbBr_{2.85}Cl_{0.15}$	$MAPbBr_{2.46}Cl_{0.54}$	$MAPbBr_{2.1}Cl_{0.9}$
Size: L×W (mm×mm)	8×6	9×8	9×7
OIHPs single crystal	$MA_{0.57}FA_{0.43}PbI_3$	$MA_{0.31}FA_{0.69}PbI_3$	$MA_{0.26}FA_{0.74}PbI_3$
Size: L×W (mm×mm)	7×5	9×7	10×9
OIHPs single crystal	$MA_{0.11}FA_{0.89}PbI_3$	$MA_{0.86}FA_{0.14}PbBr_3$	$MA_{0.76}FA_{0.24}PbBr_3$
Size: L×W (mm×mm)	4×4	10×10	7×7
OIHPs single crystal	$MA_{0.63}FA_{0.37}PbBr_3$	$MA_{0.45}FA_{0.55}PbBr_3$	
Size: L×W (mm×mm)	4×4	4×4	

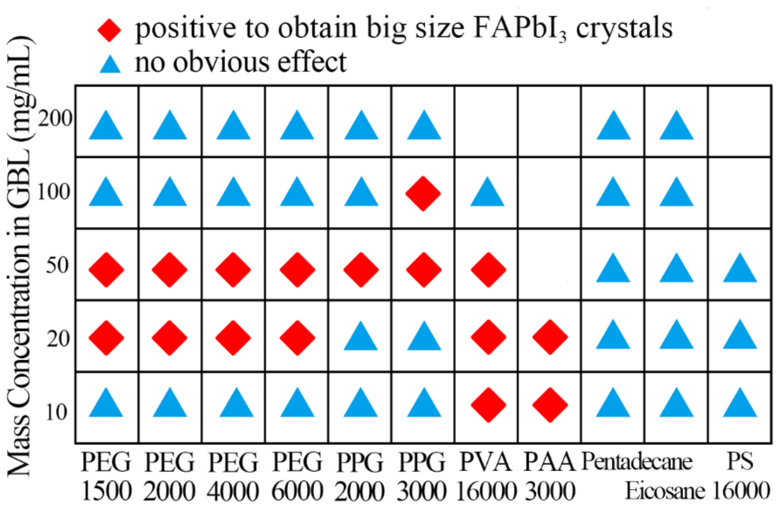

Supplementary Figure 1. Adding different polymers/additives into $FAPbI_3$/GBL growth solution with different molecular weights and quantities, the big size $FAPbI_3$ crystals may be obtained or not in the growth process. The diamonds represent positive effect on preparing big size $FAPbI_3$ crystals and the triangles represent no obvious effect. The blanks indicate the polymer could not reach this concentration in GBL.

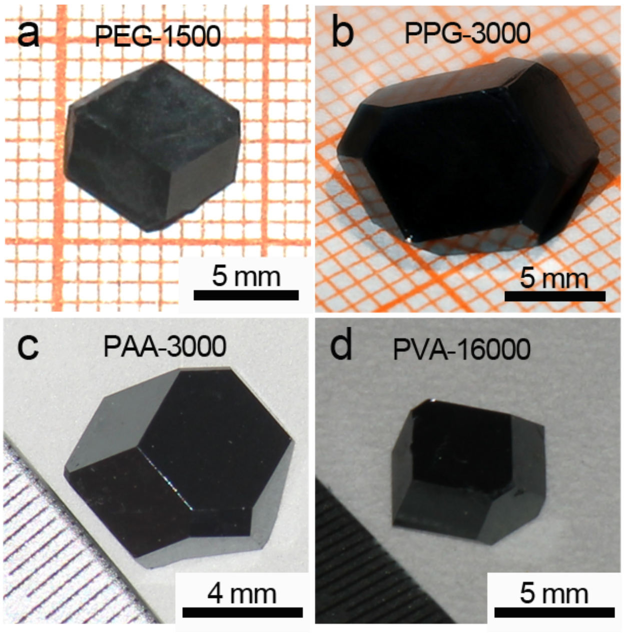

Supplementary Figure 2. The photos of as-prepared $FAPbI_3$ single crystals when adding different polymers into the precursor solutions: (a) PEG-1500, (b) PPG-3000, (c) PAA-3000 and (d) PVA-16000.

Supplementary Note 1

Black phase α-$FAPbI_3$ crystals of 1-2 millimeters in size and ill-defined shapes could be obtained without adding polymers. As shown in **Supplementary Fig. 1 and Fig. 2**, larger size $FAPbI_3$ crystals with regular geometry shapes could be grown when adding polyethylene glycol (PEG), polypropylene glycol (PPG), polyacrylic acid (PAA) or polyvinyl alcohol (PVA) of appropriate amounts and molecular weights to perovskite precursor solutions. However, other additives in comparison, such as pentadecane, eicosane, and polystyrene (PS), would not enhance the size or quality of perovskite crystals. Especially, the pentadecane could cause the needle-like yellow crystals easy to grow.

Supplementary Table 2. The information of the PPG, PEG, PAA, PVA and other additives

Type	Polymer/additive	Brand	Average Molecular Weight/Molecular Weight	Purity
poly ethylene glycol	PEG-1500	Aladdin	1500	Analytical Purity
	PEG-2000	Aladdin	2000	Analytical Purity
	PEG-4000	Aladdin	4000	Analytical Purity
	PEG-6000	Aladdin	6000	Analytical Purity
poly propylene glycol	PPG-2000	Aladdin	2000	Analytical Purity
	PPG-3000	Aladdin	3000	Analytical Purity
	PPG-400	Macklin	400	Analytical Purity
polyacrylic acid	PAA-3000 (50% solution)	Aladdin	3000	Analytical Purity
polyvinyl alcohol	PVA-16000	Aladdin	16000	Analytical Purity
pentadecane	Pentadecane	Macklin	212	Analytical Purity
eicosane	Eicosane	Macklin	283	Analytical Purity
polystylene	PS-16000	Aladdin	16000	Analytical Purity

Supplementary Table 3. A more detailed description of the growth processes for each type of OIHPs single crystals.

OIHPs	C_{OIHPs} (g/mL)	Solvent	Polymer	$C_{Polymer}$ (g/mL)	Temperature (°C)
$FAPbI_3$	0.75	GBL	PPG-3000	0.05	90
$FAPbBr_3$	0.5	GBL/DMF (*v*:*v* = 1:1)	PEG-1500	0.05	60
$MAPbI_3$	0.75	GBL	PPG-3000	0.03	95
$MAPbBr_3$	0.6	DMF	PPG-3000	0.01	62
$MAPbCl_3$	0.5	DMSO	PPG-3000	0.1	25
$CsPbBr_3$	0.35	DMSO	PPG-3000	0.08	25
$MAPbI_{2.1}Br_{0.9}$	0.75	GBL/DMF (*v*:*v* = 1:1)	PPG-3000	0.02	90
$MAPbI_{1.6}Br_{1.4}$	0.75	GBL/DMF (*v*:*v* = 1:1)	PPG-3000	0.1	90
$MAPbI_{0.12}Br_{2.88}$	0.3	GBL/DMF (*v*:*v* = 1:1)	PPG-3000	0.1	90
$MAPbBr_{2.85}Cl_{0.15}$	0.55	DMF	PPG-3000	0.01	60
$MAPbBr_{2.46}Cl_{0.54}$	0.40	DMF	PPG-3000	0.01	60
$MAPbBr_{2.1}Cl_{0.9}$	0.37	DMF	PPG-3000	0.01	60
$MA_{0.57}FA_{0.43}PbI_3$	0.75	GBL	PPG-3000	0.06	90
$MA_{0.31}FA_{0.69}PbI_3$	0.75	GBL	PPG-3000	0.06	90
$MA_{0.26}FA_{0.74}PbI_3$	0.75	GBL	PPG-3000	0.1	85
$MA_{0.11}FA_{0.89}PbI_3$	0.75	GBL	PPG-3000	0.1	85

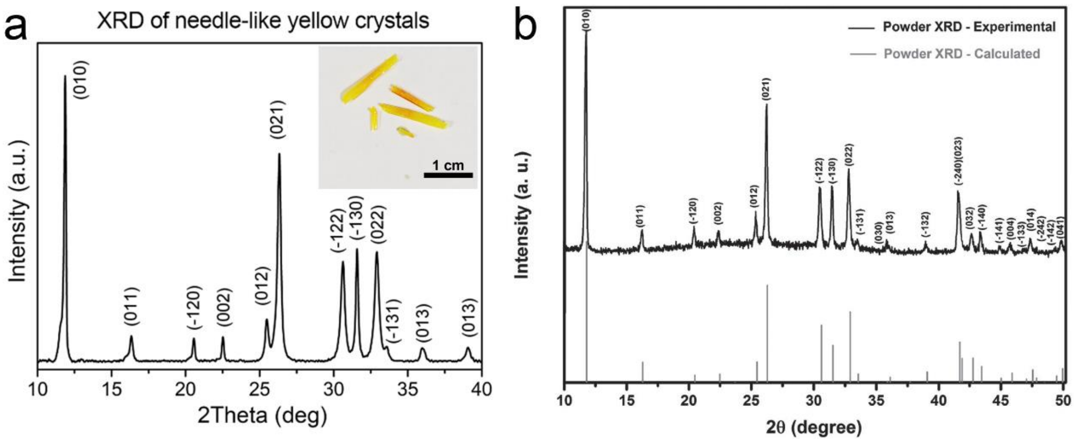

Supplementary Figure 3. (a) The powder XRD pattern of the needle-like yellow crystals from the $FAPbI_3$/GBL growth solution. (b)The experimental and calculated powder XRD patterns of δ-phase $FAPbI_3$ reported in literature. [1]

Supplementary Note 2

As reported in literature, the needle-like yellow crystals is very easy to appear during the crystallization of α-$FAPbI_3$. [1] The powder XRD pattern of the needle-like yellow crystals was shown in **Supplementary Fig. 3** and we consider that the yellow crystals were δ-$FAPbI_3$.

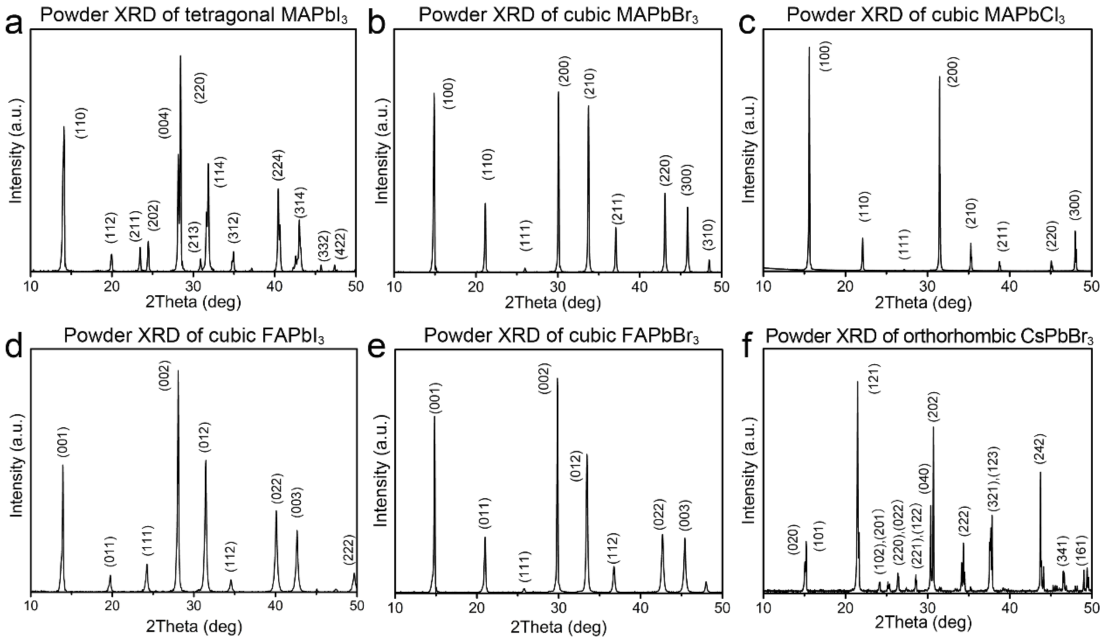

Supplementary Figure 4. The powder X-ray diffraction patterns of **a**, $MAPbI_3$, **b**, $MAPbBr_3$, **c**, $MAPbCl_3$, **d**, $FAPbI_3$, **e**, $FAPbBr_3$ and **f**, $CsPbBr_3$ powders obtained from the corresponding single crystals and the patterns are indexed to be pure perovskite phases compared with the XRD patterns reported in literatures. [2-6]

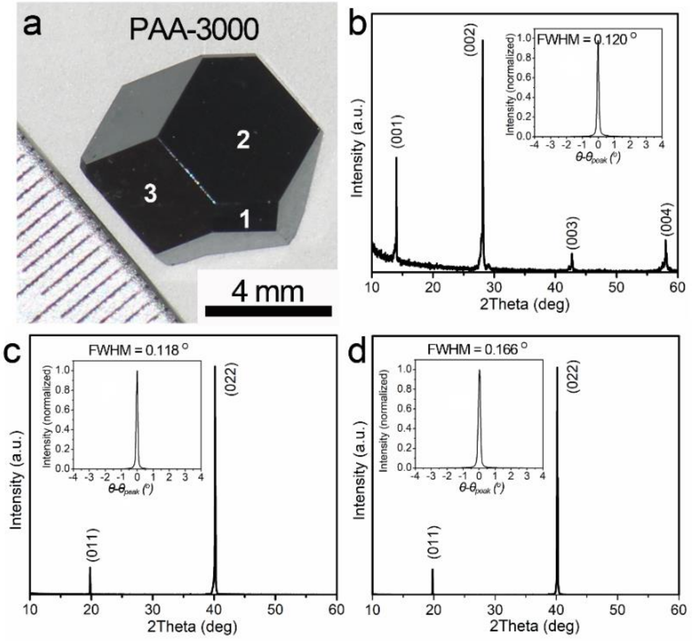

Supplementary Figure 5. (a) The $FAPbI_3$ single crystal that the crystallization process is assisted by PAA-3000. (b-d) The XRD patterns of $FAPbI_3$ single crystal on 1, 2, 3 facets and the corresponding X-ray rocking curves for the (001), (001) and (011) planes, respectively.

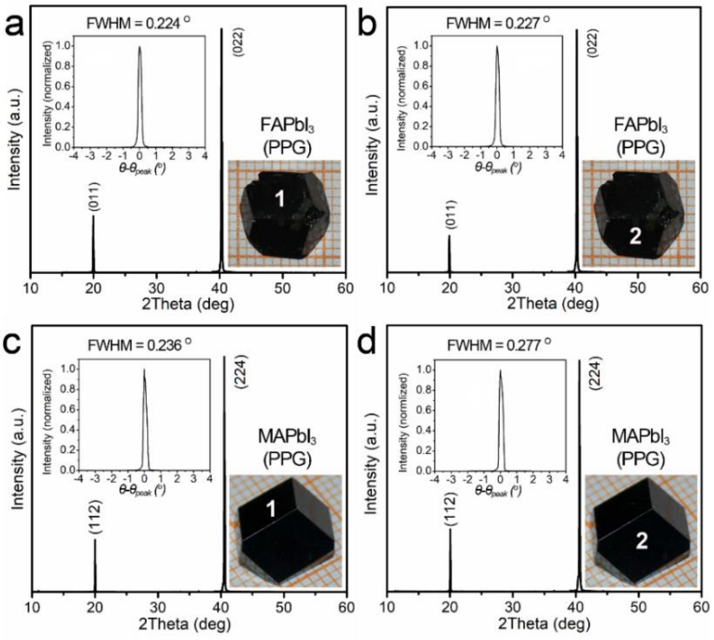

Supplementary Figure 6. (a-d) The XRD patterns of $FAPbI_3$ and $MAPbI_3$ single crystals on different facets and the corresponding X-ray rocking curves for the (011) planes of $FAPbI_3$ and the (112) planes of $MAPbI_3$. The crystallization process is assisted by PPG-3000.

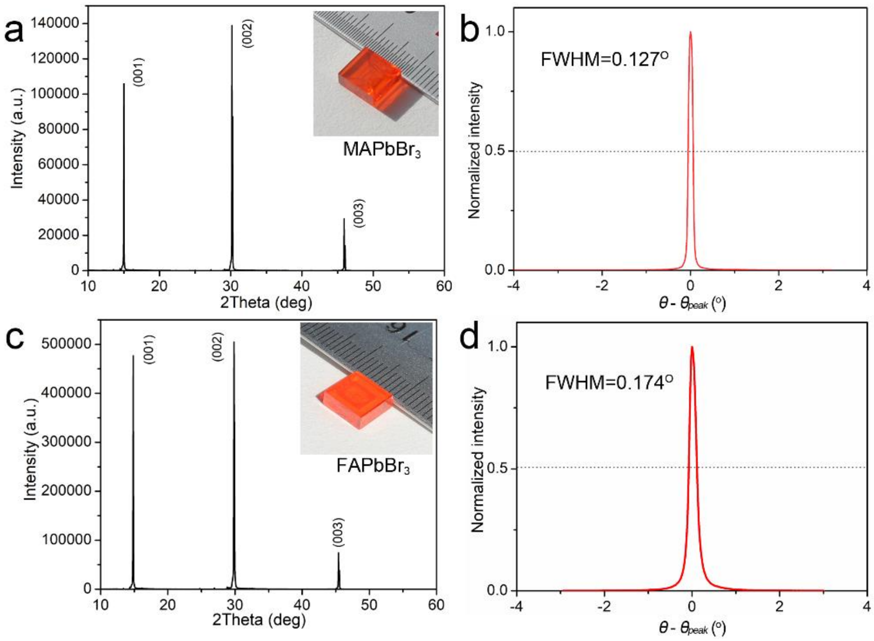

Supplementary Figure 7. (a-d) The XRD patterns of $MAPbBr_3$ and $FAPbBr_3$ single crystals and the corresponding X-ray rocking curves for the (001) planes of $MAPbBr_3$ and $FAPbBr_3$.

Supplementary Note 3

The X-ray rocking curves of several single crystals are shown in **Supplement Fig. 5, 6, 7**. The FWHM values of the $FAPbI_3$, $MAPbI_3$, $FAPbBr_3$ and $MAPbBr_3$ single crystalline samples are measured as 0.118°, 0.236°, 0.174° and 0.127°, respectively. This indicates that the single crystals are of high crystallinity.

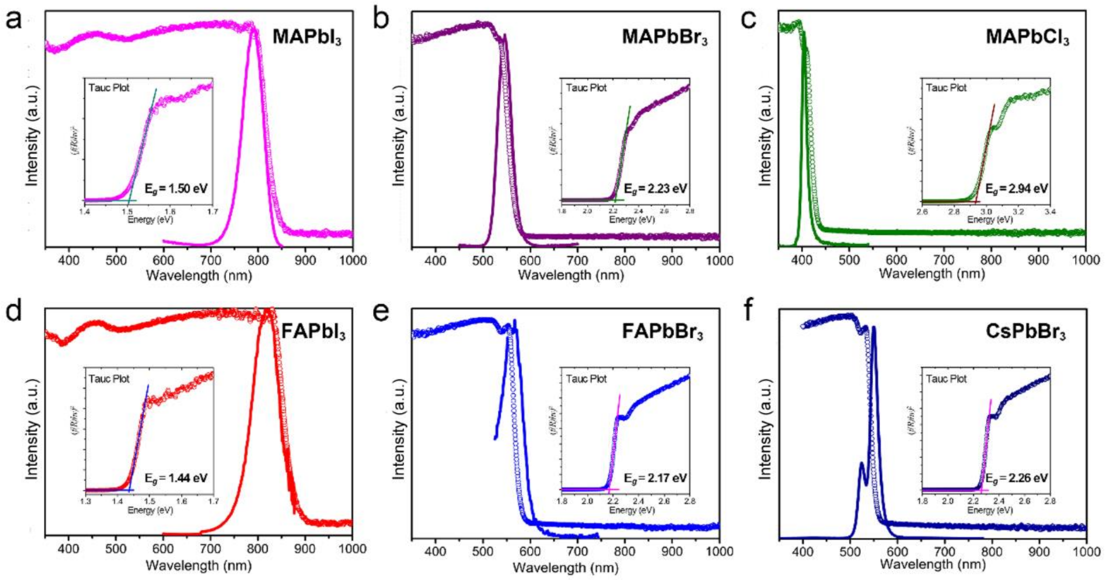

Supplementary Figure 8. UV-vis-NIR absorption spectra, Tauc plot, and photoluminescence (PL) emission (excited at 375 nm) spectra of **a**, $MAPbI_3$, **b**, $MAPbBr_3$, **c**, $MAPbCl_3$, **d**, $FAPbI_3$, **e**, $FAPbBr_3$ and **f**, $CsPbBr_3$.

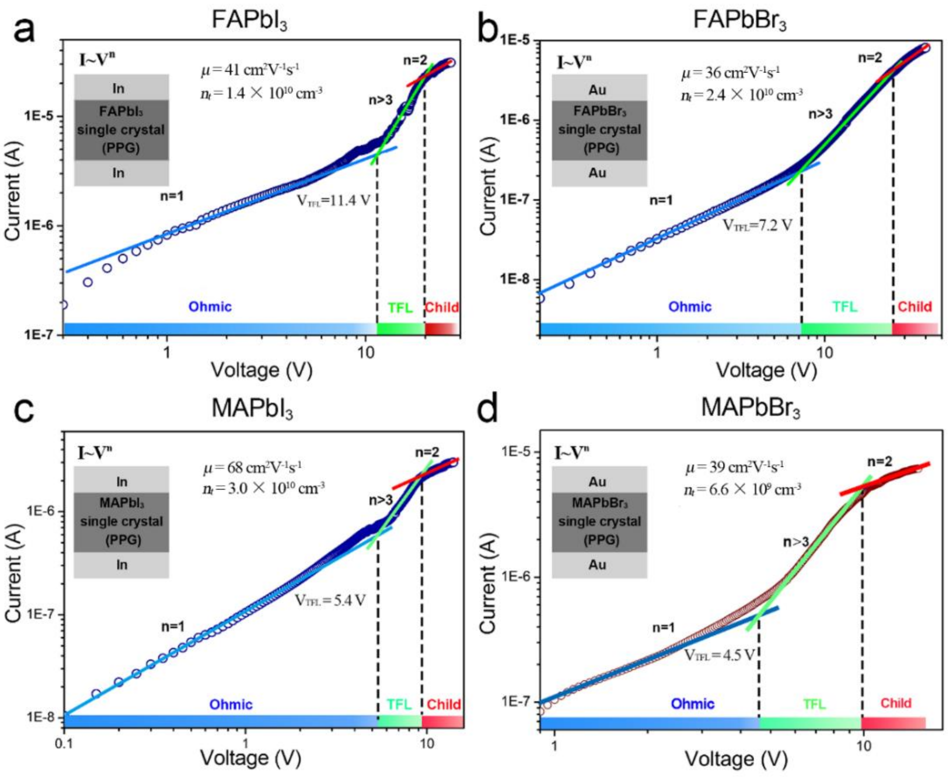

Supplementary Figure 9. Current-voltage responses of **a**, $FAPbI_3$, **b**, $FAPbBr_3$, **c**, $MAPbI_3$, and **d,** $MAPbBr_3$ single crystals, respectively.

Supplementary Note 4

The dark current-voltage (*I*-*V*) traces are measured and the carrier mobilities (μ) and the trap densities (n_{traps}) are estimated. All profiles in **Supplementary Fig. 9** show three different dependence regions ($I \sim V^n$): a linear ohmic region ($I \sim V^1$, blue lines) at a low bias, a trap filling region starting at V_{TFL} ($I \sim V^n$, $n > 3$, green lines), and a Child's region at a high bias ($I \sim V^2$, red lines). The space-charge-limited current (SCLC) technique was applied to measure the trap density and the hole carrier mobility of perovskite single crystal.[7] Two indium or gold electrodes were coated onto the sample to form a hole-only device, respectively. And a dark current measurement was carried out with a Keithley 2400 source meter. Second, we estimate the carrier lifetime τ from the time-dependent photoluminescence measurements. The carrier diffusion length is calculated by combining the carrier lifetime with mobility: $L_D = (\mu\tau k_B T/e)^{1/2}$, k_B is the Boltzmann constant and T is the sample temperature in Kelvin.

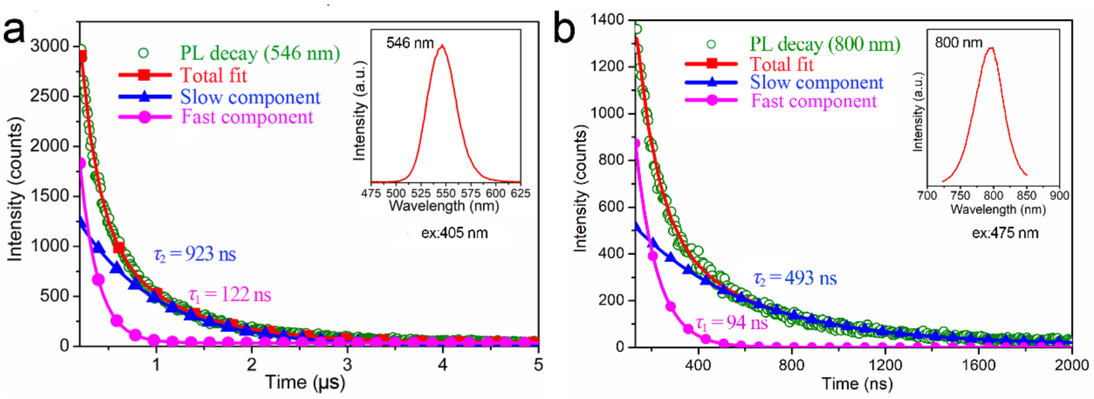

Supplementary Figure 10. Steady and transient state photoluminescence (PL) is measured using a 405 and 475 nm excitation wavelengths, respectively. PL decay curves of a, $MAPbBr_3$ b, $MAPbI_3$.

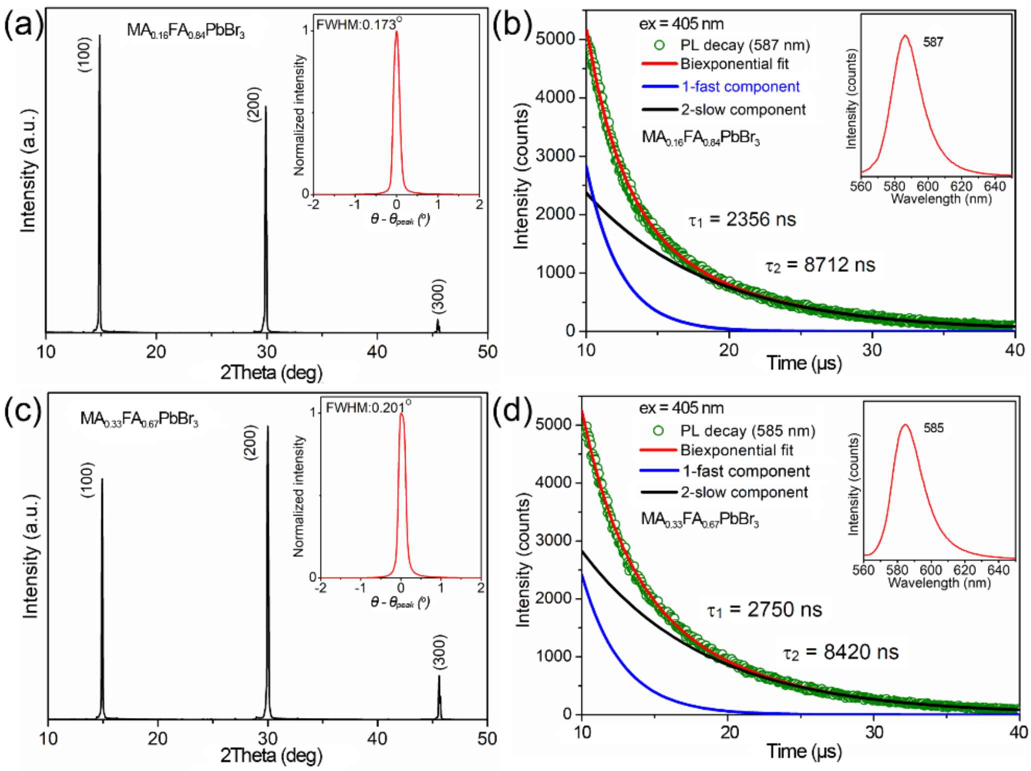

Supplementary Figure 11. (a, c) The XRD patterns of $MA_{0.16}FA_{0.84}PbBr_3$ and $MA_{0.33}FA_{0.67}PbBr_3$ single crystals and the corresponding X-ray rocking curves for the (001) planes. (b, d) PL time-decay trace of $MA_{0.16}FA_{0.84}PbBr_3$ and $MA_{0.33}FA_{0.67}PbBr_3$ crystals after 405 nm excitation.

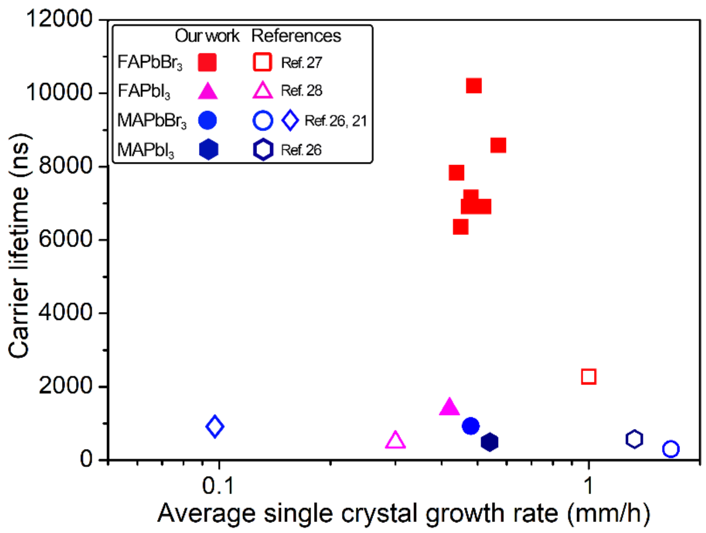

Supplementary Figure 12. The correlations between the carrier lifetimes and the average single crystal growth rates by different methods. [**Ref. 21, 26-28 in Main text**] The average single crystal growth rates (characteristic lengths, in mm/h) are calculated from the crystal size (mm) and the time of crystallization process (h).

Supplementary Table 4. Summary of high-quality $FAPbBr_3$ single crystals performance parameters in our work

No.	Carrier lifetime (ns)		Size (mm)	Growth time (h)	Average Growth rate (mm^3/h)	Average Growth rate (mm/h)
	fast component	slow component				
1	2493	10199	5×5×2	10	~ 5	~ 0.5
2	2704	8579	8×8×2.5	13.5	~ 12	~ 0.59
3	2557	7834	10×10×3	22	~ 14	~ 0.45
4	2065	6646	6.5×5.5×2	12	~ 6	~ 0.54
5	2337	6352	10×9×3	22	~ 12	~ 0.46
6	1946	6917	6×6×2	12	~ 6	~ 0.5
7	3839	7162	7×6×2.5	15	~ 7	~ 0.47

Supplementary Note 5

According to the references, we collected the parameters of OIHPs single crystal size and growth time. The average crystal growth rate can be estimated by the final crystal volume (mm^3/h) as shown in **Fig. 2d** in Main text. For example, the 10 mm $FAPbBr_3$ single crystal could be obtained in one day with the help of polymer. The average single

crystal growth rate is ~14 mm^3/h and the carrier lifetime is 7834 ns. In our work, the average single crystal growth rate of $FAPbI_3$, $MAPbBr_3$ and $MAPbI_3$ are also over 10 mm^3/h. Thus, high quality OIHPs single crystals could be prepared with high growth rate.

Supplementary Table 5. Summary of high-quality perovskite single crystals performance parameters in references.

OIHPs	Carrier lifetime (ns)		Size (mm)*	Growth time	Average Growth rate (mm^3/h)	Average Growth rate (mm/h)	Ref. in Main text
	fast component	slow component					
$MAPbBr_3$	132	897	47×41×14	20 days	~ 56	~ 0.1	21
$FAPbBr_3$	687	2272	~4×4×1	~4 h	~ 4	~ 1.0	27
$FAPbI_3$	32	484	~5×3×1	~ 10 h	~ 2	~ 0.6	28
$MAPbI_3$	18	570	~4×3	~ 3 h	~ 11	~ 1.3	26
$MAPbBr_3$	28	300	~5×4	~ 3 h	~ 7	~ 1.7	26
$MAPbI_3$	22	1032					25
$MAPbBr_3$	41	357					25
$FAPbI_3$	91	839	~4				29
$MAPbI_3$	7	146	~4				29
$MAPbBr_3$		997	~10				30

* The sizes marked with a ~ in this column are estimated from the figures in the corresponding references.

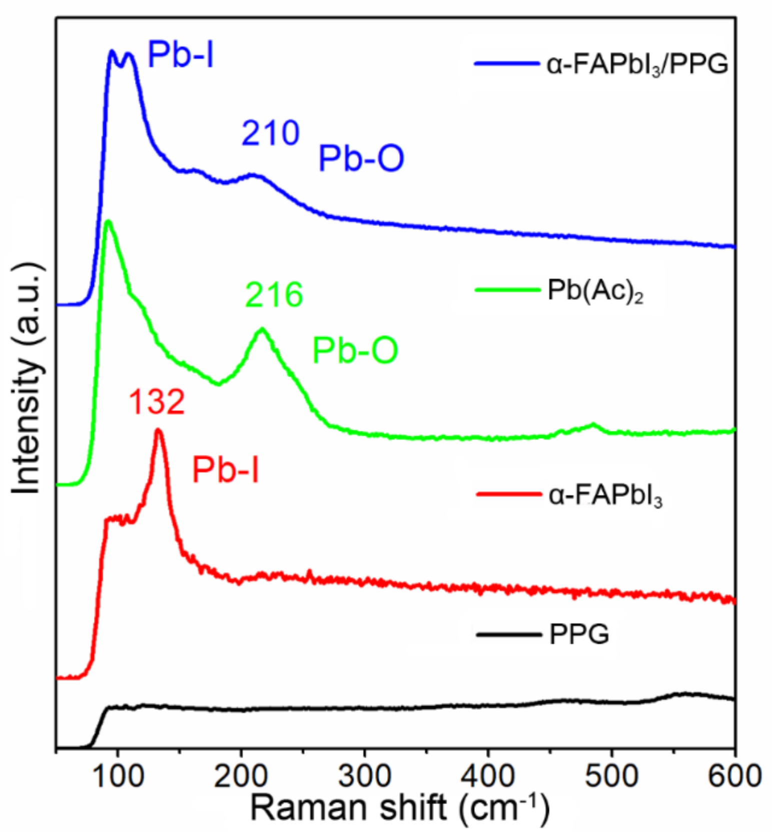

Supplementary Figure 13. Raman spectra of PPG-3000 (black), α-$FAPbI_3$ powders (red), $Pb(Ac)_2$ powders (green), α-$FAPbI_3$/PPG-3000 powders (blue) using a 532 nm wavelength laser.

Supplementary Note 6

The Raman spectra of PPG-3000, α-$FAPbI_3$ powders, $PbAc_2$ powders, and $FAPbI_3$/PPG-3000 powders are shown in **Supplementary Fig. 13.** There is no peak in the Raman spectra of pure PPG-3000 at the range of 50-600 cm^{-1}. The peak at 132 cm^{-1} of α-$FAPbI_3$ powders corresponds to the Pb-I bonding [1]. The Pb-O bonding of $Pb(Ac)_2$ is observed at 216 cm^{-1} [8]. For the $FAPbI_3$/PPG-3000 powders, a new peak appears at 210 cm^{-1} compared with the Raman spectrum of the α-$FAPbI_3$ powder. The new peak is assigned to Pb-O bond [8] which corresponds to the Raman spectrum of $PbAc_2$ sample.

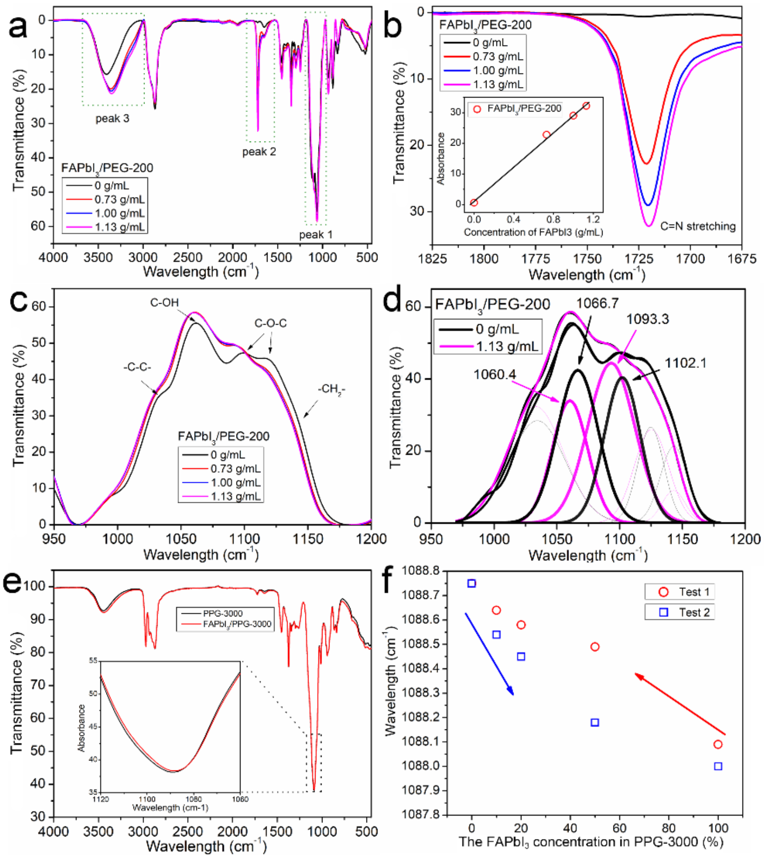

Supplementary Figure 14. (a) The IR spectra of $FAPbI_3$ dissolved in PEG-200. (b) The band near 1720 cm^{-1} in the IR spectra. (c, d) The band near 1100 cm^{-1} in the IR spectra as measured, and with the deconvoluted components. (e) The IR spectra of PPG-3000 and $FAPbI_3$/PEG-3000 solution. (f) The band near 1100 cm^{-1} in the IR spectra with different concentration of $FAPbI_3$. The concentration is described in the relative percentage of $FAPbI_3$ from the saturate solution.

Supplementary Note 7

The α-$FAPbI_3$/PEG-200 solution and α-$FAPbI_3$/PPG-3000 solution with different α-$FAPbI_3$ concentrations are measured by FTIR, respectively (**Supplementary Fig. 14**). The C-O-C band (~1100 cm^{-1}) and C-O-H band (~1060 cm^{-1}) [9] shift towards lower frequency, due to the formation Pb-O coordination bonds.

As shown in **Supplementary Fig. 14c, d** and **Supplementary Table 6**, after dissolving $FAPbI_3$ powder into PEG-200 (1.13 g/mL), the 1102.1 cm^{-1} peak shifts to a lower frequency of 1093.3 cm^{-1}, which can be assigned to the ν(C–O–C)$_{trans}$.

Furthermore, the 1066.7 cm^{-1} peak shifts to a lower frequency of 1060.4 cm^{-1}, which is assigned to the ν(C–O–H). As reported in literature, the shift in the peak towards lower frequency for PEG is attributed to the binding of C-O-C and C-O-H groups with metal ions, which reduces the electron density on O and weakens the C-O bond in turn, leading to a lowered vibration frequency. [10]

Supplementary Table 6. Parameters of the single components obtained after deconvolution of the band near 1100 cm^{-1} in liquid PEG-200 and liquid PEG-200/$FAPbI_3$ (1.13 g/mL).

	PEG-200 (cm^{-1})	α-$FAPbI_3$/PEG-200 (cm^{-1})
ν(C–C)	1034.4	1032.9
ν(C–O–H)	1066.7	1060.4
ν(C–O–C) trans	1102.1	1093.3
ν(C–O–C) gauche	1124.7	1125.2
δ(–CH_2–)	1143.2	1143.0

Supplementary Note 8

We also used the PPG-3000 as the solvent to dissolve the $FAPbI_3$ powder. It was found that the solubility of $FAPbI_3$ in PPG-3000 is low, which does not exceed 0.1 g/mL. We first added excess $FAPbI_3$ powders into the PPG-3000 and stirred for 12 hours, and then filtered it to get a saturated $FAPbI_3$/PPG-3000 solution. The $FAPbI_3$/PPG-3000 saturated solution was diluted by PPG-3000 to obtain different concentration of $FAPbI_3$/PPG-3000 solution for FTIR measurements. As shown in **Supplementary Fig. 14 e, f,** the experiment results are similar with the above-mentioned results of PEG-200.

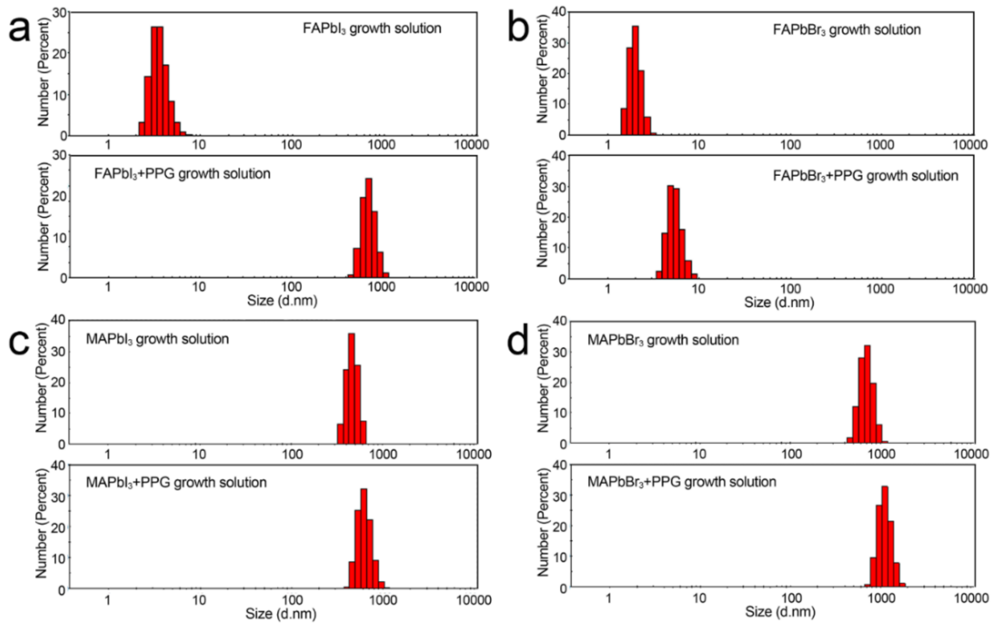

Supplementary Figure 15. The DLS particle size distribution by number: **a**, the $FAPbI_3$/GBL and $FAPbI_3$/(GBL+PPG-3000), **b**, the $FAPbBr_3$/(GBL+DMF) and $FAPbBr_3$/(GBL+DMF+PPG-3000), **c**, the $MAPbI_3$/GBL and $MAPbI_3$/(GBL+PPG-3000) and **d**, $MAPbBr_3$/DMF and $MAPbBr_3$/(DMF+PPG-3000) growth solutions by a laser particle size analyzer.

Supplementary Note 9. The detail of electrochemical measurements

Two platinum electrodes were used as test electrodes and were inserted into the precursor organic solution for the EIS testing. The solutions were tested at a scanning frequency range of 2 MHz to 100 Hz. Based on the equivalent circuit model, the R_s (ohmic resistance of solution) can be calculated, which corresponds to the solution impedance moduli at high frequency end (|Z|). The results show that the R_s (ohmic resistance) represents the solution resistance based on the equivalent circuit model. The solution resistance is in accord with the ionic concentrations in a similar solution which could be used as a parameter of solute consumption at a fixed temperature. The EIS measurements were performed every ten minutes during the crystallization process at 90 ^oC. Further analysis reveals that the R_s can be obtained using the high frequency resistance. The resistances of growth solutions with different concentration at 90 ^oC were tested using EIS at 10000 Hz. The fixed frequency measurements have the potential in real-time monitoring for being much faster with a full EIS scan. The solution concentration as a function of solution impedance moduli were obtained. Using the solution resistance obtained from the EIS profiles during crystallization, the real-time concentrations (C_R) of the growth solution could be calculated. As the original concentration of $FAPbI_3$ (C) is 0.75 g/mL, the solute consumption (C_0) could be calculated as following equation.

$$C_0 = C - C_R$$

Detailed experiment results are shown below:

(1) the Nyquist plots of growth solutions during crystallization process. Based on the equivalent circuit model, the R_s can be calculated. (**Supplementary Fig. 17**) *In situ* electrical conductance measurements have been used to investigate crystal growth, and the Nyquist plots of $FAPbI_3$ solutions (0.75 g/mL) with or no PPG-3000 (0.05 g/mL) were tested during crystallization. (**Supplementary Fig. 17**) The EIS measurements for the $FAPbI_3$/GBL solution (C_{FAPbI3} = 0.75 g/ml) and $FAPbI_3$/(GBL+PPG-3000) solution (C_{FAPbI3} = 0.75 g/mL, $C_{PPG\text{-}3000}$ = 0.05 g/mL) were performed every ten minutes.

(2) The solution resistance (R_s) as a function of growth time during the crystallization (**Supplementary Fig. 16**).

(3) The different concentrations of growth solution as a function of R_s estimated at high frequency impedance moduli (10000 Hz) (**Supplementary Fig. 18**). Then, the C_R could be calculated combined with (2) and (3).

(4) The solute consumption of $FAPbI_3$/GBL solution (C_0) with or without PPG as a function of growth time at 90 °C are shown in **Fig. 5**, **main text**.

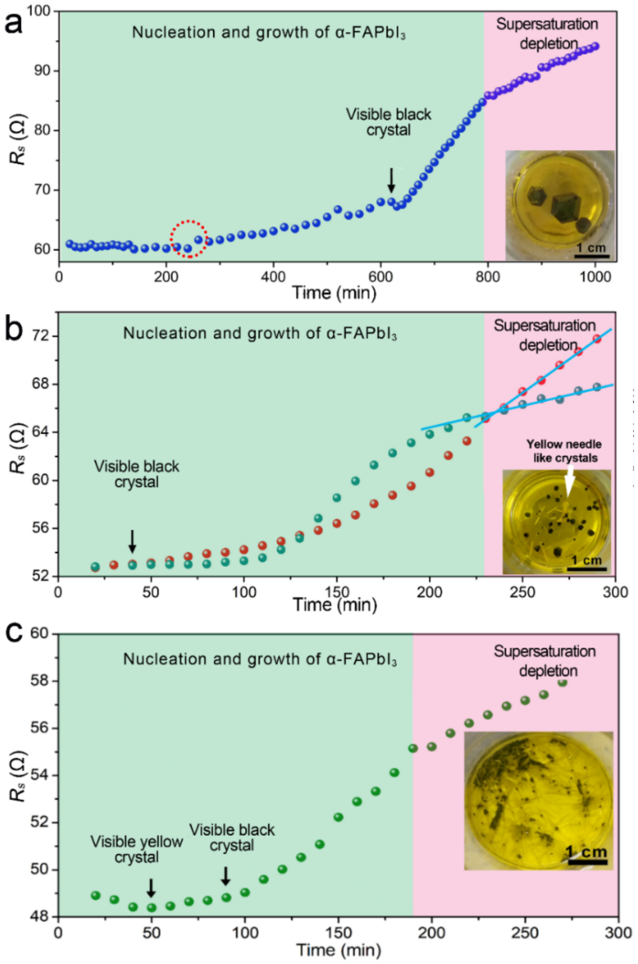

Supplementary Figure 16. (a-c) Solution resistance (R_s) as a function of growth time during the crystallization of $FAPbI_3$ single crystals: (a), $FAPbI_3$/(GBL+PPG-3000) solution (C_{FAPbI3} = 0.75 g/ mL, $C_{PPG\text{-}3000}$ = 0.05 g/mL). (b), $FAPbI_3$ /GBL solution (C_{FAPbI3} = 0.75 g/ mL). (c), $FAPbI_3$/(GBL+Pentadecane) solution (C_{FAPbI3} = 0.75 g/ mL,

$C_{Pentadecane}$ = 0.05 g/mL). Insets: The photo of crystals in growth solutions after the crystallization process.

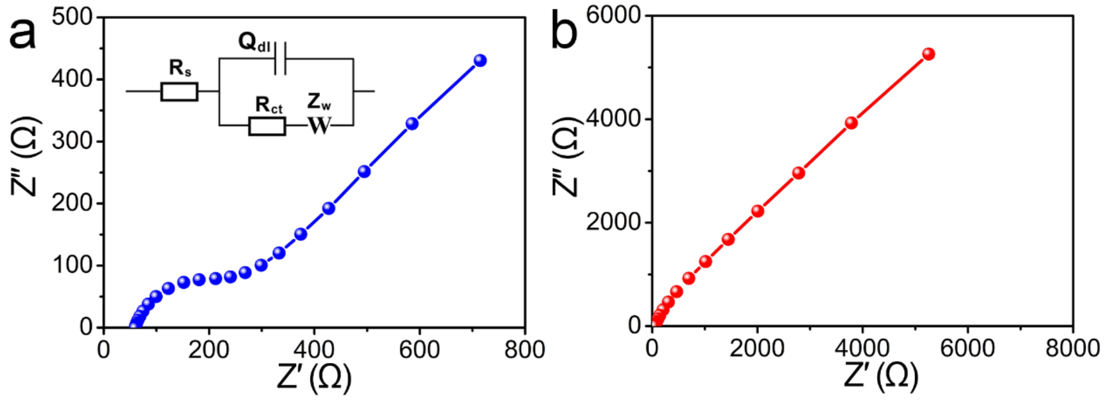

Supplementary Figure 17. (a), (b) Nyquist plots of $FAPbI_3$ solutions (0.75 g/mL) with or no PPG-3000 (0.05 g/mL) at 90 °C. (Inset: the equivalent circuit model)

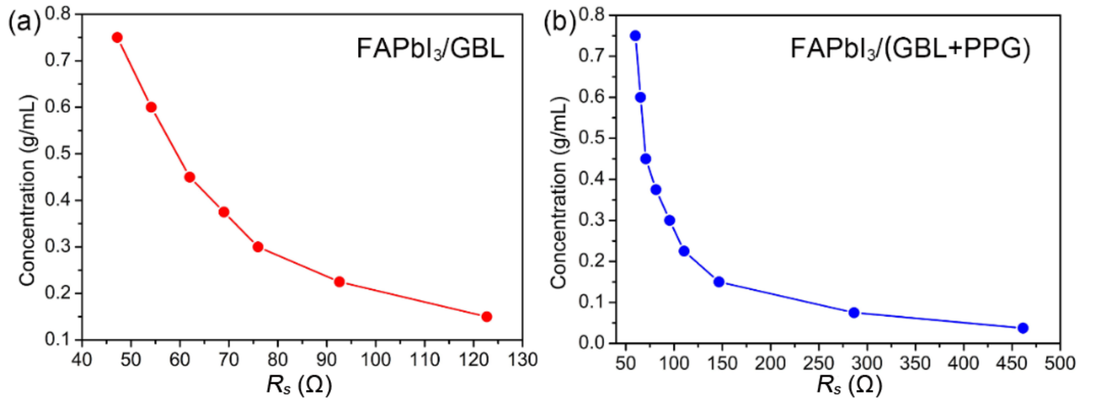

Supplementary Figure 18. (**a, b**) The concentration of $FAPbI_3$ in $FAPbI_3$/GBL solution and $FAPbI_3$/(GBL+PPG-3000) solution as a function of R_s estimated with high frequency impedance moduli at 10000 Hz. (90 °C)

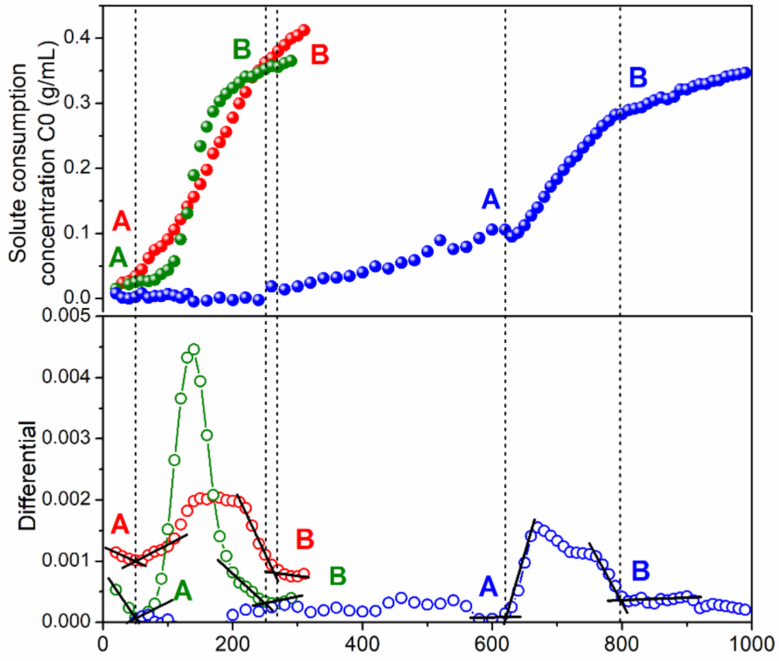

Supplementary Figure 19. The solute consumption as a function of growth time at 90 °C and the differential curves of the solute consumption. The $FAPbI_3$/GBL solution (C_{FAPbI3} = 0.75 g/mL): (1) only black crystals grow (red) or (2) yellow crystals also appear (green); The $FAPbI_3$/(GBL+PPG-3000) solution (C_{FAPbI3} = 0.75 g/ mL, $C_{PPG\text{-}3000}$ = 0.05 g/mL) that only black crystal grows (blue).

Supplementary References

1 Han, Q. F. *et al.* Single Crystal Formamidinium Lead Iodide ($FAPbI_3$): Insight into the Structural, Optical, and Electrical Properties. *Adv. Mater.* **28**, 2253-2258 (2016).

2. Liu, Y. C. *et al.* 20-mm-Large Single-Crystalline Formamidinium-Perovskite Wafer for Mass Production of Integrated Photodetectors. *Adv. Optical Mater.* **4**, 1829-1837 (2016).

3. Makhsudm, I. *et al.* High-quality bulk hybrid perovskite single crystals within minutes by inverse temperature crystallization. *Nat. Commun.* **6**, 7586 (2015).

4. Maculan, G. *et al.* $CH_3NH_3PbCl_3$ single crystals: inverse temperature crystallization and visible-blind UV-photodetector. *J. Phys. Chem. Lett*. **6**, 3781-3786 (2015).

5. Makhsud, I. *et al.* Retrograde solubility of formamidinium and methylammonium lead halide perovskites enabling rapid single crystal growth. *Chem. Commun.* **51**, 17658-17661 (2015).

6. Rakita, Y. *et al.* Low-temperature solution-grown $CsPbBr_3$ single crystals and their characterization. *Cryst. Growth Des.* **16**, 5717-5725 (2016).

7. Lampert, M. Simplified theory of space-charge-limited currents in an insulator with traps. *Phys. Rev.* **103**, 1648-1656 (1956).

8 Yang, M. M. *et al.* A Raman spectroscopic study of lead and zinc acetate complexes in hydrothermal solutions. *Geochim. Cosmtochim. Acta.* **53**, 319-326 (1989).

9 Rozenberg, M., Loewenschuss, A., Marcus., Y. IR spectra and hydration of short-chain polyethyleneglycols. *Spectrochim. Acta Part A.* **54**, 1819-1826 (1998).

10 Shameli, K. *et al.* Synthesis and Characterization of Polyethylene Glycol Mediated Silver Nanoparticles by the Green Method. *Inter. J. Molecul. Sci.* **13**, 6639-6650 (2012).
